# Natural Causes of Sudden Young Adult Deaths in Forensic Autopsies

**DOI:** 10.7759/cureus.21856

**Published:** 2022-02-03

**Authors:** Taner Daş, Aytül Buğra

**Affiliations:** 1 Morgue Department, Histopathology Unit, The Council of Forensic Medicine, Istanbul, TUR

**Keywords:** undefined death, unexplained death, young adult, sudden death, autopsy

## Abstract

Objective

The aim of this study is to define epidemiologic differences and the most common pathologies that cause nontraumatic sudden, natural death in people in the age group of 18-35 years. Identifying causes of sudden death in this age group is important for determining approaches for prevention.

Methods

We performed a descriptive statistical methodology, analysis, and interpretation using demographic and autopsy data of sudden deaths. A total of 4034 autopsies were reviewed and 66 cases of sudden death were included in this study.

Results

We identified 58 (87.9%) subjects in whom the adjudicated cause of death was of potential cardiac etiology. The most common cause of sudden young adult death was ischemic heart disease associated with the atherosclerotic coronary artery (n=24, 36.3%), followed by ischemic heart disease associated with nonatherosclerotic coronary artery disease and dissecting aortic aneurysm.

Conclusion

We put forth that the main cause of sudden young adult death was cardiac (87.9%) in origin. Of these cardiac causes, ischemic etiology associated with atherosclerosis was the main reason for sudden young adult deaths. In order to reduce the incidence of sudden young adult deaths, major efforts should be directed to prevent atherosclerosis in this age group.

## Introduction

The definition of sudden death varies [1]. Sudden death is defined by the World Health Organization as deaths occurring within 24 hours of the onset of symptoms [2]. It can also be described as sudden, unexpected, clinically unexplained deaths [1]. In the study by Ecart et al., sudden death was defined as an event that resulted in death or fatal life support within one hour after collapse, or unexpected death in the absence of a witness, in the absence of a known or suspected condition that may predispose to fatal disease [3].

Sudden death can be seen in all age groups, including infants, children, young people, adults, and the elderly. Advanced age, low or high body mass index, hypertension, diabetes mellitus, sedentary lifestyle, smoking, unhealthy diet, and stress have been reported as risk factors for sudden death [4]. Sudden death in young adults is rare, with an incidence of 0.8-6.2 per 100,000 in various studies, mostly of cardiac origin [5]. Knowing the origin and causes of sudden death is critical in preventing sudden death. Despite advances in identifying causes of sudden death, recommendations for screening young, healthy-appearing adults have remained unchanged for decades [3].

Due to the nature of sudden death, it is not possible to reach an accurate diagnosis without an autopsy. The absence of postmortem studies will make the diagnosis of sudden death causes very difficult [6]. When macroscopic findings are not evident, determining the cause of death can become complex. Despite extensive macroscopic, microscopic, and toxicological investigations, approximately 5-10% of cases will be unexplained and will be classified as sudden unexpected deaths, usually defined as death from a presumed arrhythmia. This rate varies between 30-50% in the young population. However, from a medical point of view, an unidentified etiology has dangerous clinical consequences; these unexplained deaths could potentially be due to an inherited heart disease that puts family members at risk [7]. Therefore, investigation of sudden death cases with autopsy studies is very important for the sudden death approach and treatment processes.

The aim of this study is to reveal the causes of sudden death and their incidence by retrospectively examining 66 sudden death cases between the ages of 18-35 whose autopsy was performed in the Council of Forensic Medicine Morgue Department.

This study was presented as an oral presentation at the First International 17th National Forensic Sciences Congress, Turkey (Online) on November 12-15, 2020 [8].

## Materials and methods

We conducted a definitive introductory study with permission from the Council of Forensic Medicine Scientific Research and Education Committee using the demographic and autopsy data of sudden death in 2017 in the Council of Forensic Medicine, Morgue Department. The study was approved by the Council of Forensic Medicine Scientific Research and Education Commission, Istanbul, Turkey (Number 21589509/498).

Of the 4034 deaths in the age range of 18-35 years, 66 cases of sudden young adult death that were autopsied and histopathologically examined were retrospectively re-examined. Autopsy and histopathology reports were reviewed and the findings, age, gender, and cause of death were noted. Sudden death was defined for the purpose of our study as sudden, unexpected, and nontraumatic deaths that develop within the first 24 hours from the onset of symptoms. In cases where the timing of the onset of symptoms is unknown, particularly in forensic autopsies, sudden, unexpected, and non-traumatic deaths, regardless of the time constraints are also defined as sudden death. Cases whose cause of death was suicide, poisoning, accident, drowning, and trauma were excluded, according to the definition of sudden death. Age, gender, height, weight, body mass index, and causes of death were categorized. Young adult cases between the ages of 18-35 that resulted in sudden death were included in this study, and cases outside this age range were not included in the study.

Statistical analysis

Statistical analyses were performed using Microsoft Excel 2016 for Mac (Microsoft Corp., Redmond, Washington, United States), taking into account the frequencies of the parameters and descriptive analyses (mean, minimum-maximum).

## Results

We examined 66 cadavers with sudden death between the ages of 18 and 35 from a total of 4034 cases autopsied at the Morgue Department of the Forensic Medicine Institution in 2017. Of these cases, 66.7% (n = 44) were male and 33.3% (n = 22) were female. The mean age of the cases was 29.17. The height range was 1.52-1.94 m (average 1.69 m) and the weight range was 26-132 kg (average 74.7 kg). The body mass index range was found to be 9.6-47 kg/m2 (mean 25.7 kg/mt2) (Table 1). 

**Table 1:** Age, height, weight, and body mass index of sudden young adult deaths

	Age (years)	Height (mt)	Weight (kg)	Body Mass Index (kg/mt^2^)
Minimum-Maximum	18-35	1.52-1.94	26-132	9.6-47
Mean	29.17	1.69	74.7	25.72

Cardiac origin was the cause of sudden death in 87.9% (n = 58) of 66 cases (Table 2).The most common non-cardiac causes of sudden natural death of young adults were pneumonia (n = 6, 9.1%), followed by pancreatitis (n = 1, 1.5 %) and peritonitis (n = 1, 1.5%) (Table 2).

**Table 2:** Causes of sudden young adult deaths

Cause of sudden young adult deaths	Number (n)	Percentage (%)
Cardiac	58	87.9
Pneumonia	6	9.1
Pancreatitis	1	1.5
Peritonitis	1	1.5
TOTAL	66	100

Of the cases associated with sudden cardiac death, 69% (n = 40) were male. The average age of sudden cardiac death in the 18-35 age group was 29.3 years (Table 3).

**Table 3:** Age, height, weight, and body mass index of sudden cardiac young adult deaths

	Age	Height (mt)	Weight (kg)	Body Mass Index (kg/mt^2^)
Minimum-Maximum	18-35	1.52-1.94	47-132	17.5-47
Mean	29.3	1.71	77	26.3

The most common cause of sudden death in young adult death was ischemic heart disease (n = 39, 59%), followed by dissecting aortic aneurysm (n = 7, 10.6%), cardiac valvular disease (n = 5, 7.6%), congenital heart disease (n = 3, 4.5%), cardiac conduction system abnormalities (n = 1, 1.5%), cardiomyopathy (n = 1, 1.5%), and cardiac hydatid cyst (n = 1, 1.5%) (Table 4). Sudden young adult deaths with ischemic heart disease were mostly associated with atherosclerotic coronary artery disease (n = 24, 36.3%) (Table 4).

**Table 4:** Causes of sudden young adult deaths (n=100)

Causes of sudden young adult deaths	Number (n)	Percentage (%)
Ischemic Heart Diseases	39	59
1) Atherosclerotic	24	36.3
a. Myocardial Infarction	2	3
b. Complicated Plaque (Thrombosis)	8	12
c. Noncomplicated Plaque	14	21.3
2) Nonatherosclerotic	15	22.7
a. Normal coronary artery	11	16.7
b. Bridging of coronary artery	2	3
c. Chronic Diseases	2	3
Dissecting Aortic Aneurysm	7	10.6
Valvular Diseases	5	7.6
Congenital Heart Diseases	3	4.5
Tetralogy of Fallot	1	1.5
Hypoplasia of coronary artery	1	1.5
Abnormality of coronary sinüs	1	1.5
Conducting system abnormality	1	1.5
Cardiomyopathy	1	1.5
Myocarditis	1	1.5
Cardiac Hydatid Cyst	1	1.5
Pneumonia	6	9.1
Peritonitis	1	1.5
Pancreatitis	1	1.5
TOTAL	66	100

The most important cause of sudden death in this age group was due to cardiac disease (n = 58, 87.9%) (Table 2). The most important cardiac cause of sudden young adult death was ischemic heart disease (n = 39), which was associated with atherosclerotic coronary artery disease in 61.5% (n = 24) of ischemic cases. Ischemic heart disease caused by nonatherosclerotic reasons corresponds to 38.5% (n=15) cases of ischemic heart disease. The nonatherosclerotic ischemic causes were classified as normal coronary artery (n = 11, 28.2%), coronary artery bridging (n = 2, 5.1%), and cases associated with chronic diseases (n = 2, 5.1%) (Table 5).

**Table 5:** Ischemic cardiac causes (n=39) of sudden young adult deaths

Ischemic cardiac causes of sudden young adult deaths	Number (n)	Percentage (%)
Atherosclerotic	24	61.5
Nonatherosclerotic	15	38.5
a. Normal coronary artery	11	28.2
b. Bridging of coronary artery	2	5.1
c. Chronic Diseases	2	5.1

## Discussion

Sudden unexpected deaths are an important global health problem. In some studies, it is stated that 63.4% of cardiac deaths were evaluated as sudden cardiac deaths [9]. Eckart et al. reported the causes of sudden unexplained cardiac death in the population older than 18 years in their study [3]. It has been reported that arrhythmic causes are probably the main cause of sudden unexplained cardiac death in patients under 35 years of age, but atherosclerotic heart disease is the predominant cause of sudden unexplained cardiac death in persons over 35 years of age. When they evaluated their findings, they stated that to prevent sudden death among young adults under 35 years of age, they should focus on causes not related to structural heart disease and suggested starting the prevention of atherosclerotic heart disease at earlier ages than those normally considered to be at risk. Finocchiaro et al. also stated in their study that arrhythmia and coronary artery anomalies are the most important causes of sudden death in young athletes under the age of 35, while myocardial diseases are at the forefront in athletes over the age of 40 [10].

Nofal et al. reported that the majority of sudden deaths were male (56% vs 42.2%) [1], which was also confirmed in our age-limited study (66.7% vs. 33.3%). In addition, when they grouped the age distribution of sudden death cases in their study, 32.2% were infants, 5.3% were children or adolescents between the ages of 1-18, 9.9% were young adults (18-39 years old), 21% were middle-aged (40-60 years old), and 31.4% of them belonged to the elderly (over 60 years old). Since our study was limited to only the young adult age group, there is no data in this direction in our study.

Nofal et al. reported that there is a seasonal difference in sudden deaths in their study, and they reported that it was most common in spring (29.6%), followed by summer (25.1%), followed by autumn and winter (22.8% each) [1]. Katz et al. reported the highest rates in winter (31%) and autumn (25%) [11]. Contrary to other studies, the highest rate was found in autumn (27.3%) in our study, followed by spring (25.8%), summer (24.2%), and winter (22.7%).

In a study by Sanchez et al., including postmortem genetic studies, the causes of sudden death found in the group of 20 people closest to the age range in our study were: four (20%) cardiac causes (one hypertrophic cardiomyopathy, one dilated cardiomyopathy, one congenital cardiac disease, and one myocarditis), four (20%) vascular causes (three pulmonary and one digestive system), two (10%) due to respiratory tract infections, one (5%) cerebral/neurologic edema, and two (10%) other causes (one digestive and one endocrinological reason), and the cause of death in the remaining seven (35%) cases could not be explained. Cardiac causes were major causes of sudden young deaths in our study, followed by respiratory tract infection. Our findings are consistent with other studies [1,12-20].

Cardiac causes constituted the highest rate of sudden young adult death in the 18-35 age group in which we conducted our study. Atherosclerosis was the main cause of coronary artery and ischemic heart diseases. This reveals that preventive measures against atherosclerosis should be taken, perhaps starting from an earlier age, in order to prevent and reduce sudden young adult deaths. In this respect, risk factors such as high blood pressure, high cholesterol, and triglyceride levels, smoking, and other tobacco product use, insulin resistance, diabetes, and obesity, which cause atherosclerosis, will have an important role in reducing sudden death rates in society.

Limitations

Due to the retrospective nature of our study, the data could be analyzed to some extent as available in the file records. The shortcomings in the clinical findings of the cases are our most important limitation. In addition, we could not perform the genetic evaluation in our study. This is also an important limitation of our study. 

## Conclusions

This study reveals the importance of forensic autopsies in determining the causes of sudden young adult deaths. It determines that preventive efforts should be directed primarily on cardiac causes, especially atherosclerosis. Comprehensive studies carried out in the light of postmortem examinations will reveal the causes of young adult sudden death cases that may be genetically caused.
